# Vascular anatomy of the lateral meniscus with special focus on the joint capsule

**DOI:** 10.1007/s12565-024-00797-z

**Published:** 2024-08-31

**Authors:** Yutaro Natsuyama, Yuka Mitsuya, Miyuki Kuramasu, Shinichi Kawata, Tomiko Yakura, Zhong-Lian Li, Shuang-Qin Yi, Masahiro Itoh

**Affiliations:** 1 Department of Anatomy, Tokyo Medical University, 6-1-1, Shinjuku, Tokyo, Japan; 2 Department of Frontier Health Sciences, Graduate School of Human Health Sciences, Tokyo Metropolitan University, Tokyo, 116-8551 Japan

**Keywords:** Intercapsular route, Intracapsular route, Meniscus hilum, Popliteal hiatus, Popliteus muscle

## Abstract

Previous studies have reported that the lateral meniscus (LM) has two regions, the popliteal hiatus area (PH) with a scarce blood supply and the roots with an abundant one. However, the description of its vascular anatomy remains insufficient. We hypothesized that the difference in the width of the meniscus hilum (MH) affects the scarcity and abundance of blood supply to the LM. The MH is a concept proposed by us and is the only site of entrance or exit of blood vessels and nerves associated with the meniscus. The purpose of this study was to provide a structural explanation for the disparity of blood supply to the LM using the concept of MH. Sixteen knees were examined to investigate the blood supply to LM. In most areas, the femoral joint capsule (FJC) and tibial joint capsule (TJC) continued to the cranial and caudal edges of the LM, respectively. In the roots, the FJC and TJC covered the femoral and the outer-femoral surfaces. In contrast, the FJC in the PH did not attach to the cranial edge and only the TJC there did to the caudal edge of the LM. Histochemical examination showed that the blood vessels enter the LM via the MH. In the PH, the MH at the caudal edge was extremely narrow; and in the roots, the MH on the outer-femoral surfaces was wide. The results suggest that the difference in the width of the MH affected the scarcity and abundance of blood supply to the LM.

## Introduction

**Fig. 1:**
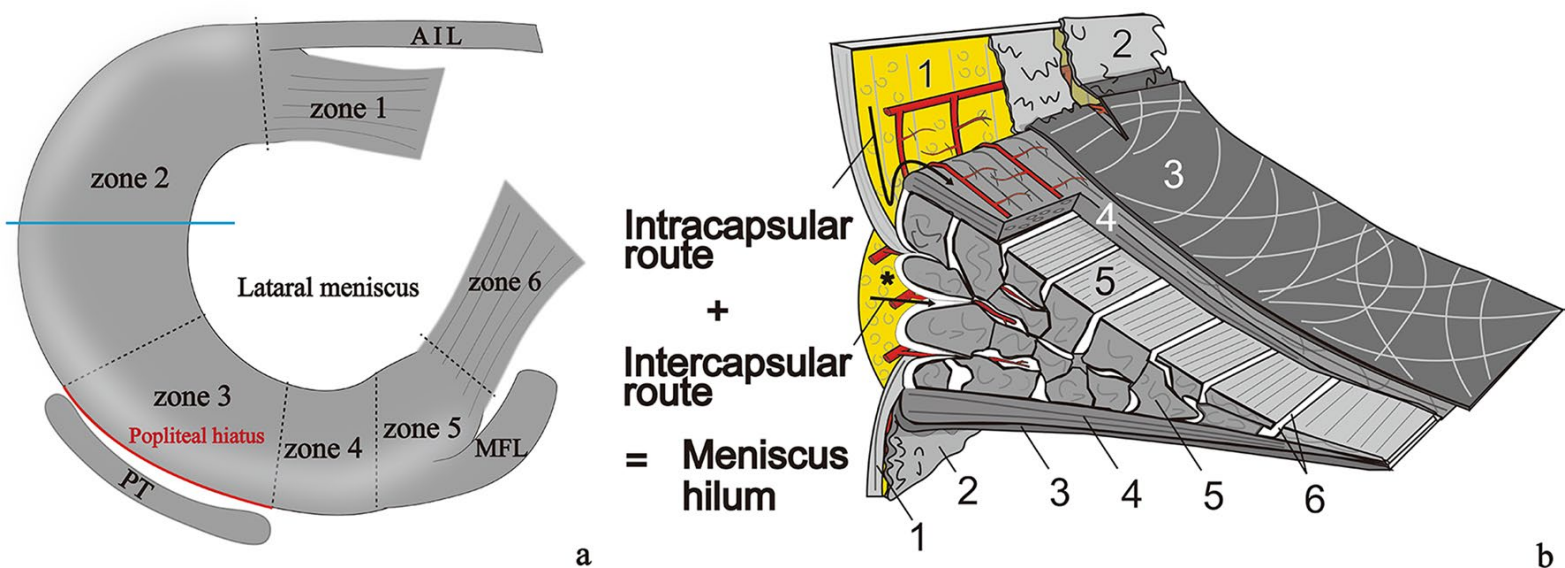
Schematic views of LM. **a** The left LM view from the horizontal plane. **b** A schematic view from the cross-section of a blue line in **a** showing a minute structure of the meniscus hilum (Natsuyama et al. 2023a). 1, fibrous layer; 2, synovial layer; 3, superficial network; 4, lamellar layer; 5, circumferential bundles; 6, tie fiber; an asterisk, peri-meniscal fat pad; *AIL* anterior intermeniscal ligament, *MFL* meniscofemoral ligament, *PT* popliteus muscle tendon

Based on the anteroposterior position, the lateral meniscus (LM) can be divided into six zones (Zdanowicz et al. 2016). These include the anterior root (zone 1), the anterolateral zone between the anterior root and the anterior border of the popliteal hiatus area (PH) (zone 2), PH (zone 3), the posteroinferior popliteomeniscal fascicle (zone 4), the ligamentous zone (zone 5), and the posterior root (zone 6) (Fig. 1a). It has been reported that blood supply is scarce in the PH (Arnoczky et al. 1982; Day et al. 1985) and abundant in the anterior and posterior horn attachments (roots) for the LM (Arnoczky et al. 1982; Day et al. 1985). However, to our knowledge, no morphologic basis for the difference has been provided. We hypothesized that this could be explained by the anatomical concept of the meniscus hilum, proposed by our study using pigs (Natsuyama et al. 2023a). The meniscus hilum is the only site of entrance or exit of blood vessels and nerves associated with the meniscus (Fig. 1b). The meniscus hilum is formed by both intracapsular and intercapsular routes (Fig. 1b). While the intracapsular route refers to the pathway between the fibrous layer and the synovial layer of the joint capsule (JC) to between the lamellar layer and the superficial network of the meniscus, the intercapsular route refers to the pathway between the femoral joint capsule (FJC) and the tibial joint capsule (TJC) to the meniscus (Fig. 1b). Thus, it was presumed that the difference in the width of the meniscus hilum affects the scarcity and abundance of blood supply to the LM.

The JC is composed of a fibrous layer and a synovial layer (Ralphs and Benjamin 1994). Although the fibrous layer is individually named by their attachment points (such as capsular ligaments), the focus of this study is not on the knee joint stability but on the blood supply, so the collagen fibers that cover the synovial layer are treated as the fibrous layer of the JC. The purpose of this study was to provide a structural explanation for the disparity in blood supply to the LM.

## Materials and methods

### Preparation of specimens

A total of 16 knees (7 right and 9 left) from 11 Japanese cadavers (6 males and 5 females; mean age at death, 81.6 years; range, 67–93 years) were used. Informed consent for education and research was acquired from the members of the Toju-kai, a body donation group of Tokyo Medical University. Study approval was obtained from the Ethics Committee of Tokyo Medical University (Approval Number: T2020-0050). The authors hereby confirm that every effort was made to comply with all local and international ethical guidelines and laws concerning the use of human cadaveric donors in anatomical research. All cadavers were fixed by arterial perfusion of 3.8% formalin and preserved in 60–70% alcohol. The study did not include cadavers with a history of knee abnormalities.

### Macroscopic arterial examinations

Four of the 16 knees were used to observe the arterial origin to the LM. In the dissection of the arteries, to clearly show the peripheral microarteries, 30 ml of a mixture of red ink and latex was injected into the femoral and deep femoral arteries with pressurization (Natsuyama et al. 2023b). After removing the skin and subcutaneous soft tissues, the arteries to the LM were dissected and photographed.

### Morphologic measurements

Twenteen of the 16 knees (including histological samples) were used for morphologic measurements. The JC, LM, and popliteus muscle were harvested as a block from the knee. The JC and LM were observed with special attention to the attachment. The total length of the LM (femoral and tibial sides, areas not covered by JC), the length of femoral anterior root to PH, and length of the PH were measured with electronic caliper (P02 110–120, As One Corp., Japan) (Fig. 3), referring to Aman et al. (2019).

### Histological and immunohistochemical examination

Eight of the 16 knees were examined for histologic measurements to assess the presence of arteries and JC attachment structures using serial coronal sections. The skin of the specimen was peeled, and the subcutaneous fat and excess muscle tissues were also carefully removed.

An LM-JC popliteus muscle complex was harvested as a tissue block. Before tissue embedding, it was re-fixed by immersion in 5% formalin for 1 week, thoroughly washed under running tap water for 4–5 h, and then decalcified in 10%EDTA (pH8.0) for 1 month, after which it was dehydrated and routinely embedded in paraffin. Five-micrometer-thick sections were cut in each zone of the LM, and the slides were stained with Hematoxylin and Eosin (HE), Masson’s trichrome, and CD31 immunohistology. CD 31 is a marker of endothelial cells of blood vessels.

The HE staining procedure was performed as follows: (1) deparaffinization and rehydration (xylene for 5 min, 3 times; 100% ethanol for 5 min, 3 times; 95% ethanol for 5 min; 85% ethanol for 5 min; 70% ethanol for 5 min; tap water), (2) hematoxylin staining (Gill’s hematoxylin applied for 1 min; rinsed with warm tap water), and (3) eosin staining and dehydration (95% ethanol 5 min; eosin Y applied for 1 min; immersed in 95% ethanol, 2 times; 100% ethanol for 5 min; and xylene for 5 min, 3 times).

The Masson’s trichrome staining procedure was performed as follows: (1) deparaffinization and rehydration (same HE stain), (2) hematoxylin staining (mordant for 30 min; tap water for 5 min; Carrazzi's hematoxylin applied for 45 min; rinsed with tap water), (3) orange G staining (0.75% orange G applied for 1 min; 1% acetic acid, 2 times), (4) Masson B (Masson B applied for 20 min), (5) phosphomolybdic–phosphotungstic acid differentiating (1 min; 1% acetic acid, 2 times), and (6) Aniline blue staining and dehydration (Aniline blue applied for 30 min; 1% acetic acid, 2 times; immersed in 100% ethanol for 5 min, 3 times; and xylene for 5 min, 3 times).

Immunohistochemistry was performed with reference to Omotehara et al. (2023): slides were deparaffinized and hydrated in xylene, ethanol, and distilled and deionized water. They were heated for 20 min at 120 ℃ in 10 mM citrate buffer pH 6.0 for antigen retrieval. Endogenous peroxide activity was quenched by immersing in 0.3% H_2_O_2_ in absolute methanol for 30 min at room temperature. After nonspecific binding of the primary antibody was blocked by incubation with Blocking One Histo (Nacalai Tesque) for 30 min at room temperature, the primary antibody, monoclonal antibody against CD31, diluted in phosphate-buffered saline containing 0.05% Tween20 (PBS-T) was reacted for 18 h at 4 °C. EnVision + System-HRP Labelled Polymer Anti-Rabbit (Agilent Dako) were used as secondary antibodies for rabbit. After washing in PBS-T, avidin-biotinylated peroxidase complex was performed slides for 30 min. EnVision rabbit for DAB (Agilent Dako) was incubated with washed slides and Gill's hematoxylin V was used as a counterstain for 5 min.

The slides were digitized with an automatic digital slide scanner (Panoramic MIDI II, 3DHISTECH, Hungary) at × 20 magnification. Images were merged and measured using Adobe Illustrator 2024 (Adobe Inc., USA).

### Statistical analysis

Each data set is represented by its mean ± standard deviation (SD). Significant differences were determined using a paired-matched *t* test with the level of significance set at a *p* value of < 0.05.

## Results

### Arterial origin of lateral meniscus

**Fig. 2:**
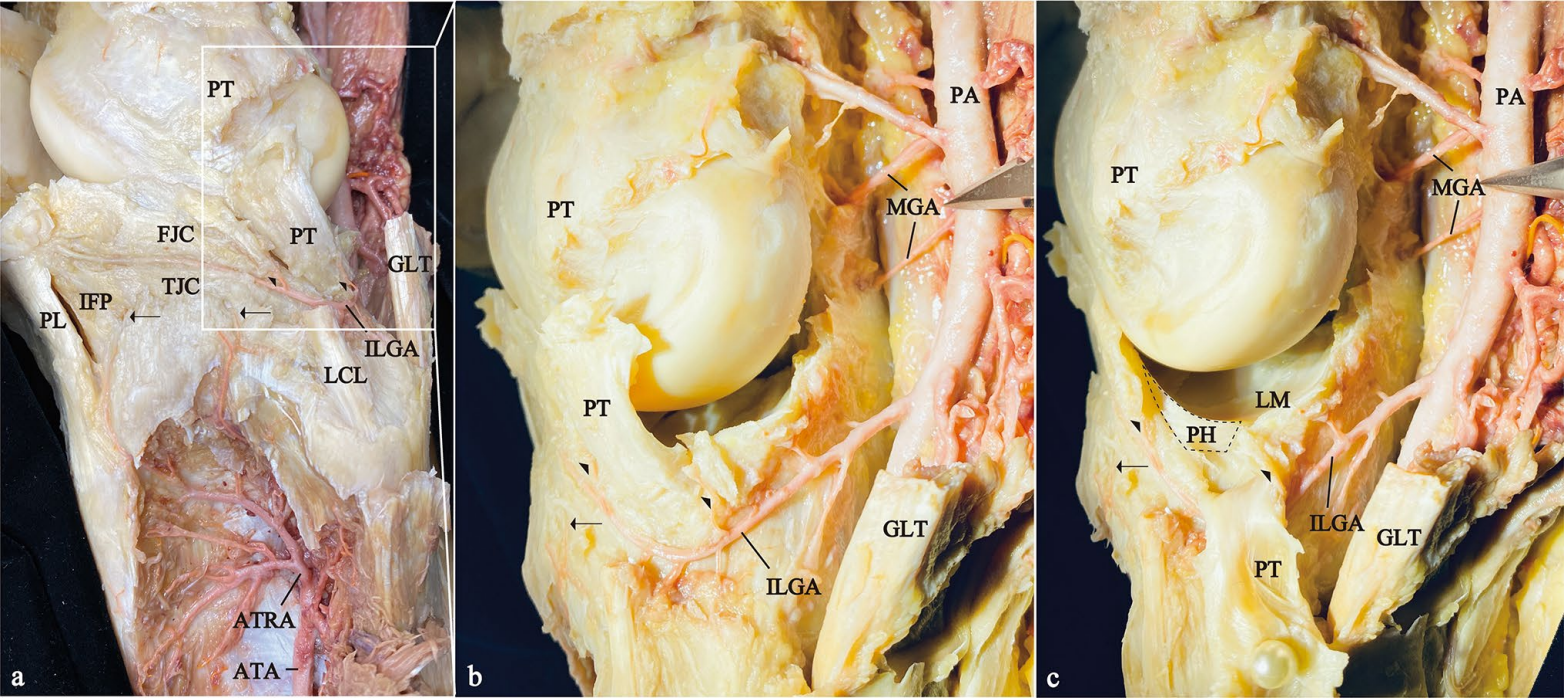
Photographs of the arterial origin of the left LM. **a** Lataral view of the left knee with some tissue removed to show the arteries. **b** The posterior superior lateral view of the left knee. **c** And the same view in which the PT was inverted to show the arteries of the LM. *ATA* anterior tibial artery, *ATRA* anterior tibial recurrent artery, *FJC* femoral joint capsule, *GLT* gastrocnemius lateral tendon, *IFP* infrapatellar fat pad, *ILGA* inferior lateral genicular artery, *LCL* lateral collateral ligament, *LM* lateral meniscus, *MGA* middle genicular artery, *PA* popliteal artery, *PH* popliteal hiatus area, *PL* patellar ligament, *PT* popliteus muscle tendon, *TJC* tibial joint capsule; arrows, ATRA to LM; arrowheads, branches from ILGA to the front and back of popliteal hiatus

The LM nutrient arteries typically originated from the inferior lateral genicular artery and the middle genicular arteries (Fig. 2).

The inferior lateral genicular artery, which arose from the popliteal artery, passed deep to the lateral head of the gastrocnemius muscle, curved inferiorly at its intersection with the popliteus tendon, traveled anteriorly on the outer surface of the LM, and terminated at the patellar ligament.

The middle genicular arteries originated from the popliteal artery at the level of the knee joint as short branches and supplied the posterior horn and root of the LM (Fig. 2b, c).

The anterior tibial recurrent arteries, which arose from the anterior tibial artery, ran parallel to the recurrent branch of the common peroneal nerve with branches to the surrounding muscles and terminated in the inferior part of the patellar ligament (Fig. 2a). LM branches from the anterior tibial recurrent artery were observed in 2 of the 4 cases. One was distributed on the anterior horn and body (Fig. 2a), and another was distributed on the anterior horn only.

The superior lateral genicular artery, which arose from the popliteal artery, ran superficial to the lateral head of the gastrocnemius muscle and branched to the surrounding muscles and soft tissue. No branch from the superior lateral genicular artery to the LM was identified.

In addition, the PH did not receive a direct artery because of blocking the path by presence of popliteus muscle (Fig. 2c).

### Macroscopic observations of the lateral meniscus and joint capsule

**Fig. 3:**
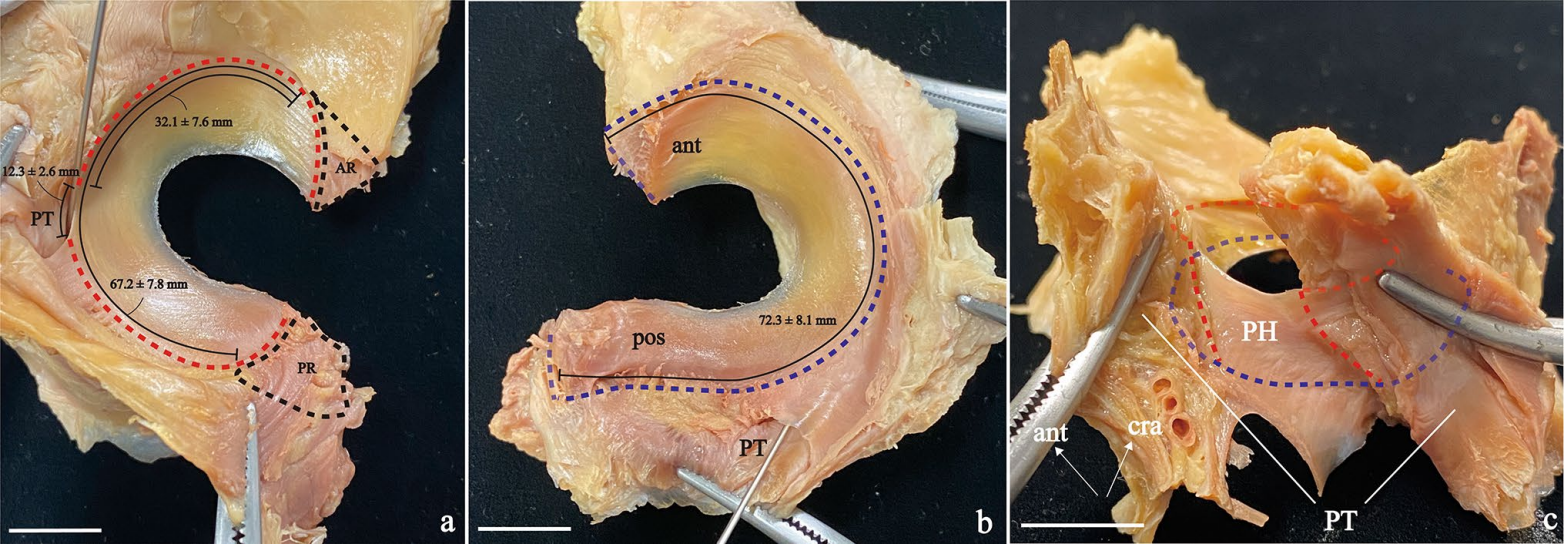
Photographs showing the attachment of the JC to the LM at left. **a** The femoral surface and **b** tibial surface of the LM (popliteal hiatus with a pin through it). JCs were pulled laterally. The red and blue dotted lines show the FJC and TJC attachment to the LM, respectively. The black dotted lines show the anterior root (AR, zone 1) and posterior root (PR, zone 6). **c** The outer surface of the left LM, to show the popliteal hiatus area, the popliteus muscle tendon was cut into two parts on the long axis. *ant* anterior, *cra* cranial, *pos* posterior, *PH* popliteal hiatus area, *PT* popliteus muscle tendon; Scale bar: 1 cm

Total length of the LM on the femoral side was 67.2 ± 7.8 mm, in which length of femoral anterior root to PH and length of PH were 32.1 ± 7.6 mm and 12.3 ± 2.6 mm, respectively (Fig. 3a, b). In contrast, total length of the LM on the tibial side was 72.3 ± 8.1 mm and significantly longer than total length of the LM on the femoral side because the femoral surface of the anterior/posterior roots was covered by FJC and TJC.

In zones 2, 4, and 5, the FJC and TJC continued to the cranial and caudal edges of the outer surface of the LM, respectively (Fig. 3). In the roots of zones 1 and 6, the FJC and TJC covered the femoral and outer surfaces (Fig. 3). In zone 3 (PH), the FJC did not continue to the cranial edge of the PH and only the TJC attached to the caudal edge of the LM (Fig. 3c).

### Histological observations of the lateral meniscus and joint capsule

**Fig. 4:**
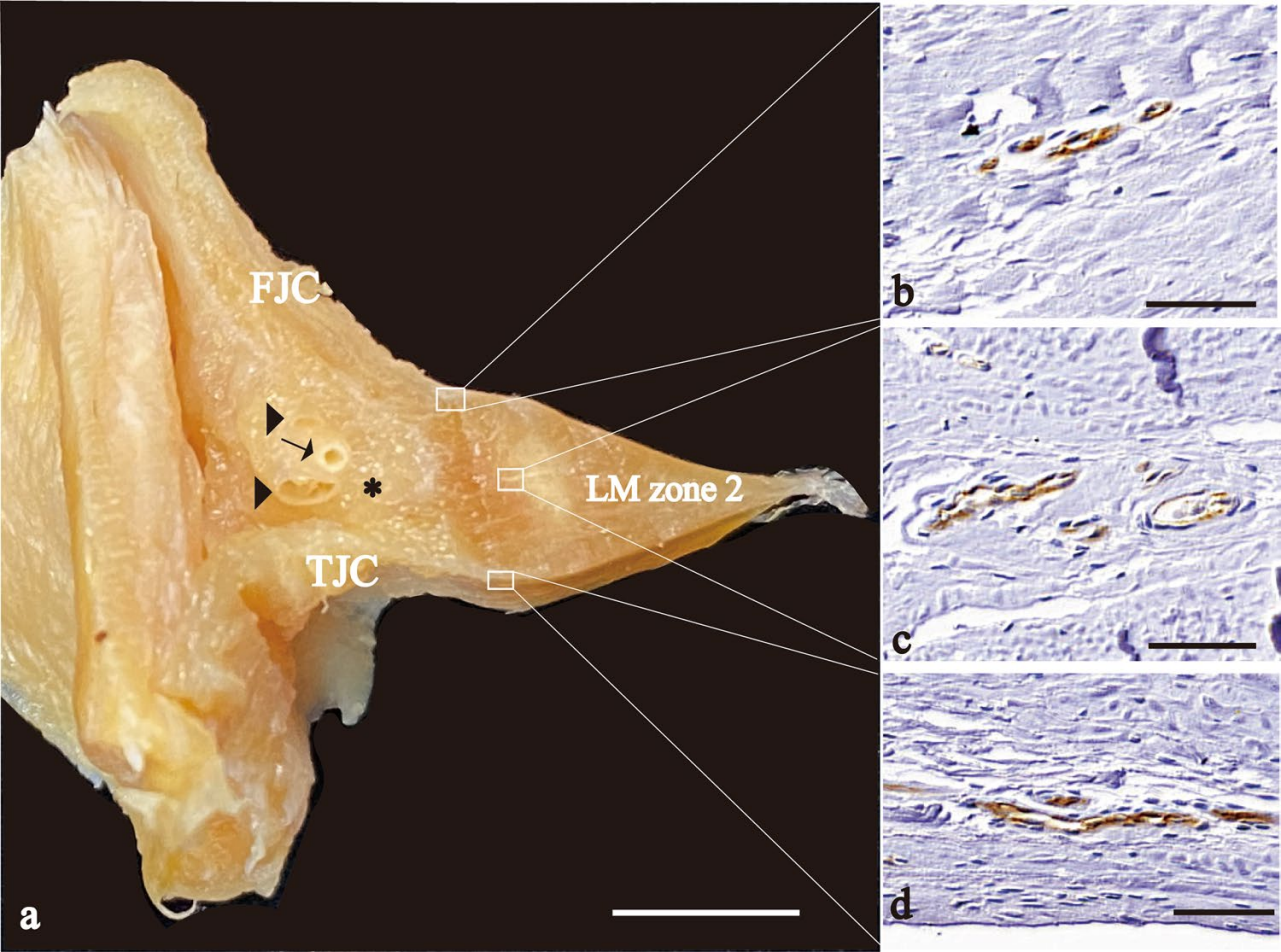
Photographs that show the vascularity of the intracapsular and intercapsular routes. **a** Frontal plane of a cross-section of the LM zone 2. **b**–**d** Immunohistochemical sections of 3 segments of (**a**) stained with CD31. **b** FJC intracapsular route; **c** intercapsular route; and **d** TJC intracapsular route. *FJC* femoral joint capsule, *TJC* tibial joint capsule; an arrow, an artery; arrowheads, veins; an asterisk, peri-meniscal fat pad; Scale bar: 5 mm (**a**), 50 μm (**b**–**d**)

In zone 2, the FJC and the TJC attached to the cranial and caudal edges of the outer surface of the LM, respectively (Fig. 4). Both JCs showed positive reactions for CD31 at the LM attachment site (Fig. 4b, d). Between the JCs, cross-sections of an artery and two veins were observed in the peri-meniscal fat pad. These blood vessels branched into the LM (Fig. 4c). Similar findings were also noted in zones 4 and 5.

**Fig. 5:**
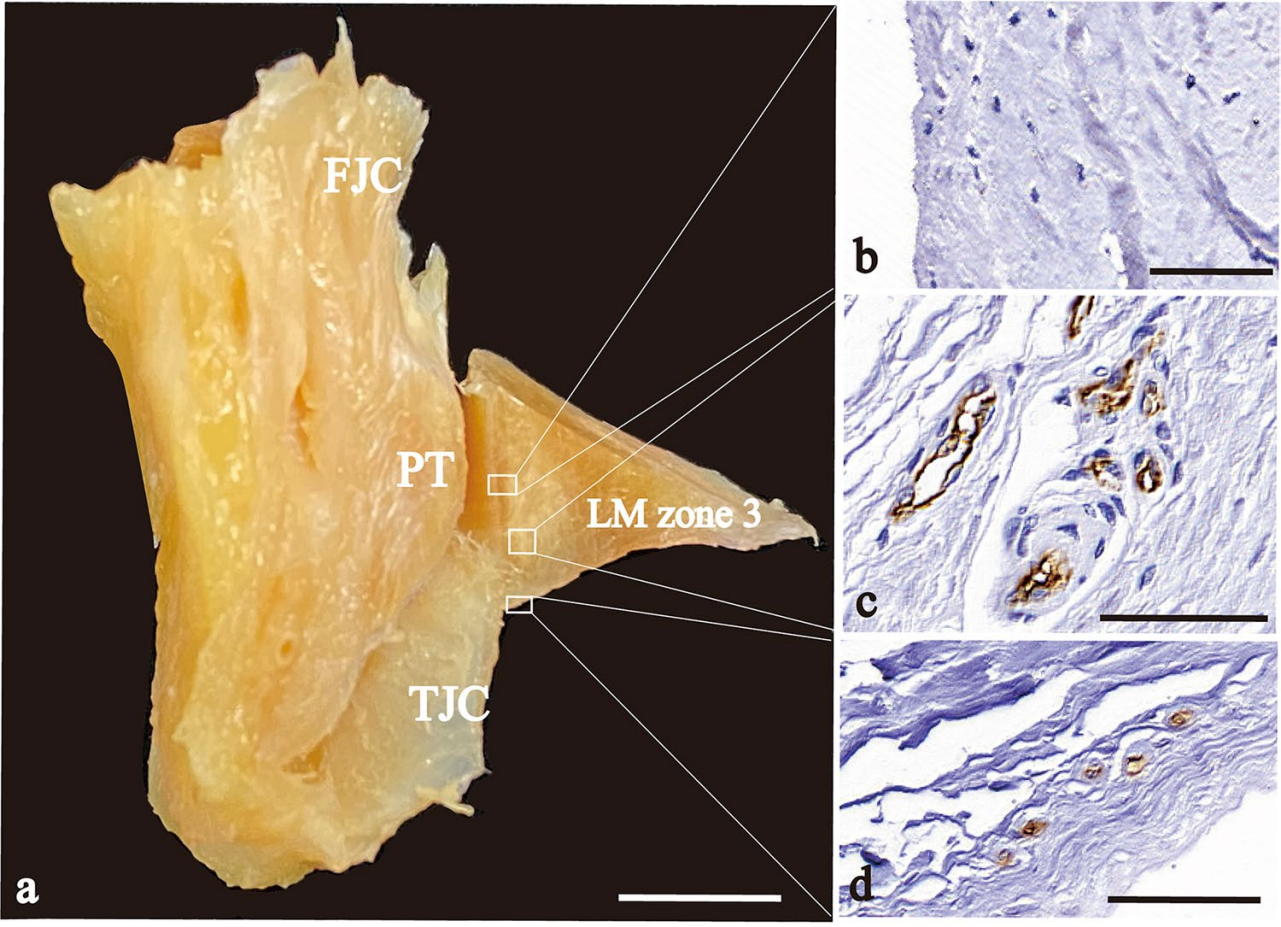
Photographs that show the vascularity of the caudal half and avascularity of the cranial half. **a** Frontal plane of a cross-section of the LM zone 3. **b**–**d** Immunohistochemical sections of 3 segments of (**a**) stained with CD31. **b** the popliteal hiatus area (no attachment of JC); **c** the extension of intercapsular route from other zones; and **d** TJC intracapsular route. *FJC* femoral joint capsule, *PT* popliteus muscle tendon, *TJC* tibial joint capsule. Scale bar: 5 mm (**a**), 50 μm (**b**–**d**)

In contrast, the TJC in the PH (zone 3) attached to the caudal edge of the outer surface and exhibited positive reactions for CD31 (Fig. 5c, d). However, in zone 3, the FJC did not attach to the LM and the cranial half of the outer surface of the LM did not exhibit a positive reaction for CD31 (Fig. 5b).

**Fig. 6:**
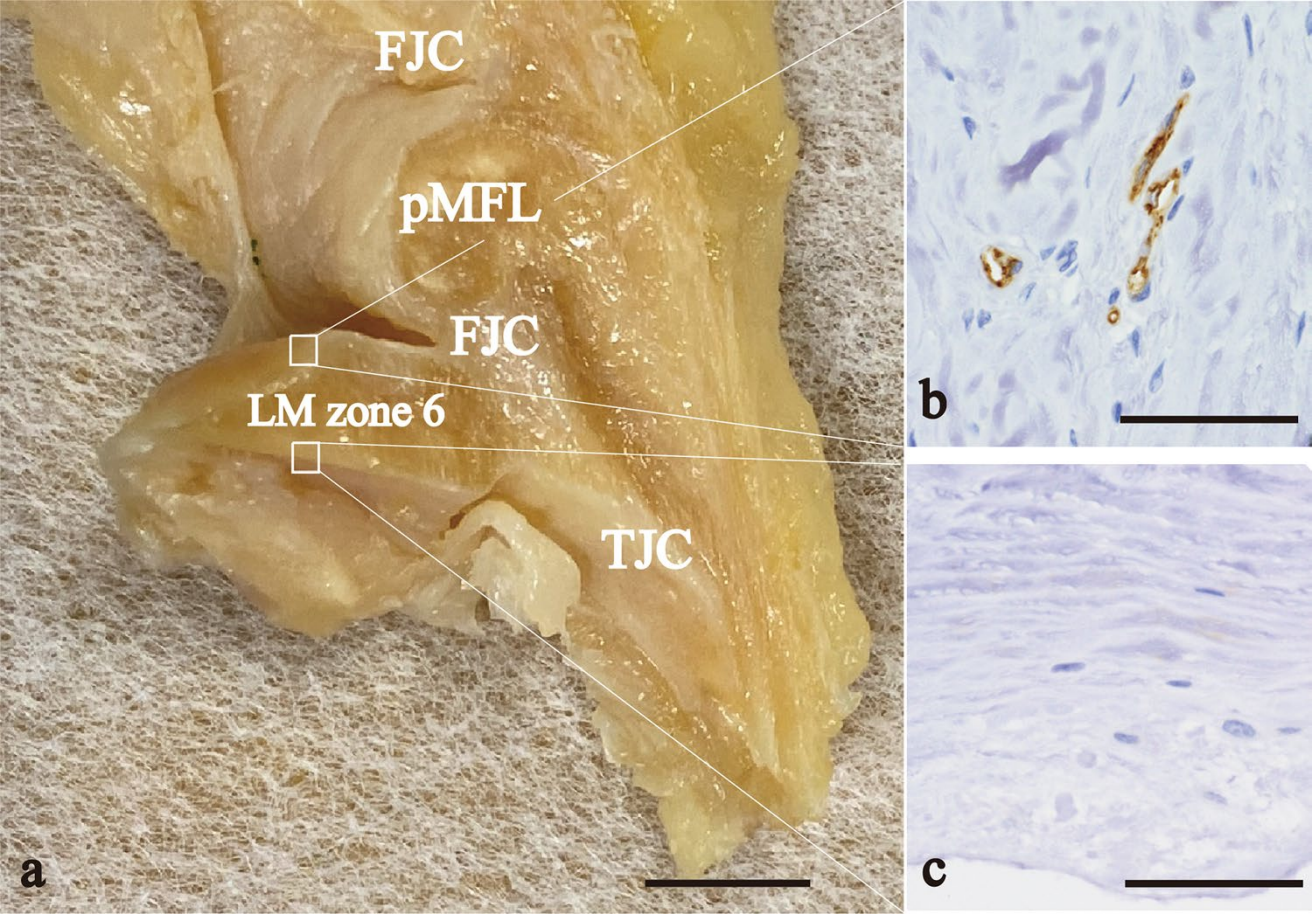
Photographs that show the vascularity of the inner part in the femoral surface and avascularity of the inner part in the tibial surface. **a** Sagittal plane of a cross-section of the LM zone 6. **b**, **c** Immunohistochemical sections stained with CD31. **b** the femoral surface of the posterior root to which the FJC attached; **c** the tibial surface of the posterior root to which the TJC did not attach. *FJC* femoral joint capsule, *pMFL* posterior meniscofemoral ligament, *TJC* tibial joint capsule. Scale bar: 5 mm (**a**), 50 μm (**b**, **c**)

In the posterior root (zone 6), the FJC and TJC attached to the outer-femoral surface and positive CD31 reactions were observed at the site (Fig. 6b). In contrast, the TJC did not attach to the tibial surface and no CD31-positive reaction was observed in the tibial surface (Fig. 6c). Similar findings were also obtained in anterior root (zone 1).

## Discussion

**Fig. 7:**
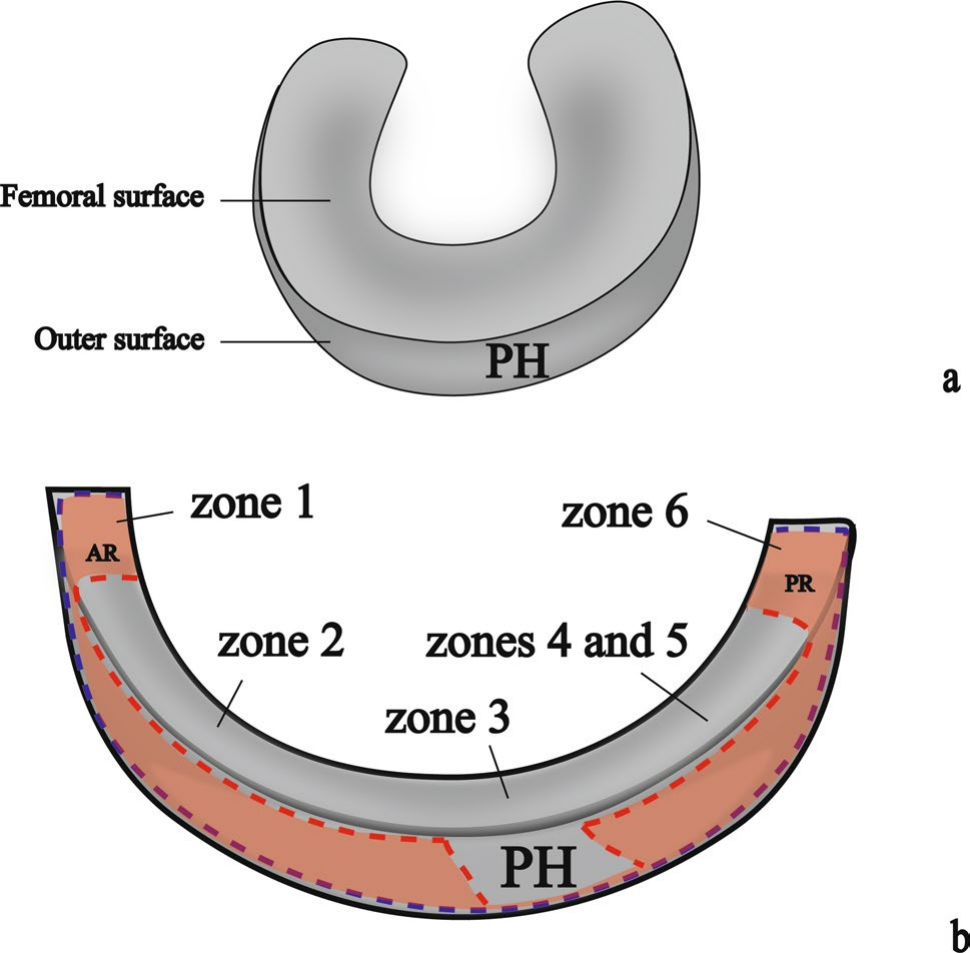
Schematic views of surfaces of LM. **a** Femorolateral view of left LM. **b** Partially unfolded anatomy of (**a**) showing the attachment of the JCs to the LM. The red and blue dotted lines show the FJC and TJC attachment to the LM, respectively. Meniscus hilum is indicated by the light red area. *AR* anterior root, *PH* popliteal hiatus area, *PR* posterior root

In this study, we evaluated the arterial origin, JC attachment, and the relationship between JC attachment and blood vessels regarding the LM. This study provided a clear perspective on the detailed arterial supply to the LM. Overall, the LM was typically supplied by both the inferior lateral genicular artery and the middle genicular artery. In two of four cases, the anterior tibial recurrent artery was supplying to LM. In histological examination, blood vessels were observed at the attachments of FJC and TJC to LM and also between both JCs. No blood vessel was noted in the area where JC did not attach. In most of the LM, the FJC and TJC attached to the cranial and caudal edges, respectively; in the PH, only the TJC attached to the caudal edge; and in the anterior and posterior roots, both JCs covered the femoral surface (Fig. 7).

The immunohistochemical evaluation showed that capillaries had entered the LM via the intracapsular or intercapsular route. This means that the concept of the meniscus hilum can be applied also in humans. The meniscus hilum, formed by the intracapsular and the intercapsular routes, functions as a transport channel between the inside and outside of the meniscus (Natsuyama et al. 2023a). Based on this concept of the meniscus hilum, the scarce and abundant blood supply to the LM can be explained. It has been reported that blood supply is scarce in the PH (Arnoczky et al. 1982; Day et al. 1985), which can be explained by narrow meniscus hilum at the intracapsular route of the TJC. That is why few blood vessels can enter the LM from the outside. Morphological observations indicated that there was no FJC attachment (approximately 12.3 mm) in the cranial edge of PH, which means that this area could not be penetrated by the blood vessels. These measurements, shown in Fig. 3, are similar to those of previous studies (Cohn and Mains 1979; Aman et al. 2019; Grassi et al. 2021; Gamble et al. 2022). Furthermore, several previous studies have reported that LM disease is associated with PH widening and edema (De Smet et al. 2001; Li et al. 2021; Heaton et al. 2024). This can be explained by a narrowing of the meniscus hilum. Widening of PH causes narrowing of the meniscus hilum, leading to reduction of the venous drainage, and the reduction of venous drainage causes edema. On the other hand, anterior and posterior roots have been reported to have abundant blood supply (Arnoczky et al. 1982; Day et al. 1985), which can be explained by the wide meniscus hilum on outer-femoral surface. This study showed that anterior and posterior roots have wide meniscus hilum because the JCs attached the femoral surface. The wide meniscus hilum allows a large number of blood vessels to enter the LM. Therefore, we consider that the concept of a meniscus hilum is useful for a better understanding of the entrance and exit of blood vessels and nerves associated with the meniscus.

The arteries to the meniscus have been described as the distribution of the knee arterial network (Kean et al. 2017; Pereira et al. 2019; Bhan 2020). In the present study, the inferior lateral genicular artery and the middle genicular arteries were observed to branch to the LM. This is consistent with previous studies (Arnoczky et al. 1982; Day et al. 1985; Gee and Posner 2021; Mameri et al. 2022), although the distribution of the superior lateral genicular artery to LM was reported in some cases (Allen et al. 1995; Gray 1999). However, we also found the anterior tibial recurrent artery branched to the LM in two cases.

This study has limitations. First, the sample size (16 knees) was relatively small. Additional samples, such as other 40 knees, are needed to quantify the JC attachment more objectively and also classify the variations of blood supply, however, we think that the present study using 16 knees is sufficient to discuss the concept of the meniscus hilum in humans. Second, the ages of the materials were skewed because the cadavers used in this study were those of elderly adults with an average age of > 66 years. To address this issue, cadavers without gross deformity or orthopedic history were used.

## Conclusions

Arterial branches entered the LM via the intracapsular and intercapsuler routes of the meniscus hilum. In most areas (except for the roots and PH), the meniscus hilum was one outer surface of the LM; in the PH, the meniscus hilum at the caudal edge was narrow; and in the anterior and posterior roots, the meniscus hilum on the outer-femoral surfaces was wide. The present study suggested that the difference in the width of the meniscus hilum affects the scarcity of blood supply to the PH and the abundance of blood supply to the roots of the LM.

## Data Availability

The data supporting the results of this study are available from the corresponding author upon reasonable request.

## References

[CR1] Allen AA, Caldwell GL, Fu FH (1995) Anatomy and biomechanics of the meniscus. Oper Tech Orthop 1:2–9. 10.1016/S1048-6666(95)80041-7

[CR2] Aman ZS, DePhillipo NN, Storaci HW et al (2019) Quantitative and qualitative assessment of posterolateral meniscal anatomy: defining the popliteal hiatus, popliteomeniscal fascicles, and the lateral meniscotibial ligament. Am J Sports Med 47(8):1797–1803. 10.1177/0363546519849933 31136201 10.1177/0363546519849933

[CR3] Arnoczky SP, Warren RF (1982) Microvasculature of the human meniscus. Am J Sports Med 10(2):90–95. 10.1177/036354658201000205 7081532 10.1177/036354658201000205

[CR4] Bhan K (2020) Meniscal tears: current understanding, diagnosis, and management. Cureus 12(6):e8590. 10.7759/cureus.8590 32676231 10.7759/cureus.8590 PMC7359983

[CR5] Cohn AK, Mains DB (1979) Popliteal hiatus of the lateral meniscus. Anatomy and measurement at dissection of 10 specimens. Am J Sports Med 7(4):221–226. 10.1177/036354657900700402 474859 10.1177/036354657900700402

[CR6] Day B, Mackenzie WG, Shim SS, Leung G (1985) The vascular and nerve supply of the human meniscus. Arthrosc J Arthrosc Relat Surg 1(1):58–62. 10.1016/s0749-8063(85)80080-3 10.1016/s0749-8063(85)80080-3 4091911

[CR7] De Smet AA, Asinger DA, Johnson RL (2001) Abnormal superior popliteomeniscal fascicle and posterior pericapsular edema: indirect MR imaging signs of a lateral meniscal tear. AJR Am J Roentgenol 176(1):63–66. 10.2214/ajr.176.1.1760063 11133540 10.2214/ajr.176.1.1760063

[CR8] Gamble JG, Abdalla AB, Meadows MG et al (2022) Radial width of the lateral meniscus at the popliteal hiatus: relevance to saucerization of discoid lateral menisci. Am J Sports Med 50(1):138–141. 10.1177/03635465211056661 34780308 10.1177/03635465211056661

[CR9] Gee SM, Posner M (2021) Meniscus anatomy and basic science. Sports Med Arthrosc Rev 29(3):e18–e23. 10.1097/JSA.0000000000000327 34398117 10.1097/JSA.0000000000000327

[CR10] Grassi A, Pizza N, Andrea Lucidi G, Macchiarola L, Mosca M, Zaffagnini S (2021) Anatomy, magnetic resonance and arthroscopy of the popliteal hiatus of the knee: normal aspect and pathological conditions. EFORT Open Rev 6(1):61–74. 10.1302/2058-5241.6.200089 33532087 10.1302/2058-5241.6.200089 PMC7845568

[CR11] Gray JC (1999) Neural and vascular anatomy of the menisci of the human knee. J Orthop Sports Phys Ther 29(1):23–30. 10.2519/jospt.1999.29.1.23 10100118 10.2519/jospt.1999.29.1.23

[CR12] Heaton DJ, Collins MS, Johnson AC, Krych AJ, Dancy ME, Tiegs-Heiden CA (2024) Retrospective evaluation of MRI findings in arthroscopically confirmed cases of hypermobile lateral meniscus. Skeletal Radiol 53(3):465–472. 10.1007/s00256-023-04433-1 37620610 10.1007/s00256-023-04433-1

[CR13] Kean CO, Brown RJ, Chapman J (2017) The role of biomaterials in the treatment of meniscal tears. PeerJ 5:e4076. 10.7717/peerj.4076 29158995 10.7717/peerj.4076 PMC5695244

[CR14] Li Z, Fan W, Dai Z et al (2021) Widening of the popliteal hiatus on sagittal MRI view plays a critical role in the mechanical signs of discoid lateral meniscus. Knee Surg Sports Traumatol Arthrosc 29(9):2843–2850. 10.1007/s00167-020-06179-y 32728789 10.1007/s00167-020-06179-y

[CR15] Mameri ES, Dasari SP, Fortier LM et al (2022) Review of meniscus anatomy and biomechanics. Curr Rev Musculoskelet Med 15(5):323–335. 10.1007/s12178-022-09768-1 35947336 10.1007/s12178-022-09768-1 PMC9463428

[CR16] Natsuyama Y, Zhang M, Yang T et al (2023a) The continuous structure of the joint capsule and meniscus in the pig knee. Anat Histol Embryol 52(5):789–797. 10.1111/ahe.12938 37306076 10.1111/ahe.12938

[CR17] Natsuyama Y, Zhang M, Yang T et al (2023b) Morphological study of the arterial supply to the menisci in pigs with special reference to creating meniscus injury model. Folia Morphol. 10.5603/FM.a2023.0041 10.5603/FM.a2023.0041 37285086

[CR18] Omotehara T, Hess RA, Nakata H, Birch LA, Prins GS, Itoh M (2023) Expression patterns of sex steroid receptors in developing mesonephros of the male mouse: three-dimensional analysis. Cell Tissue Res 393(3):577–593. 10.1007/s00441-023-03796-0 37335379 10.1007/s00441-023-03796-0

[CR19] Pereira H, Fatih Cengiz I, Gomes S et al (2019) Meniscal allograft transplants and new scaffolding techniques. EFORT Open Rev 4(6):279–295. 10.1302/2058-5241.4.180103 31210969 10.1302/2058-5241.4.180103 PMC6549113

[CR20] Ralphs JR, Benjamin M (1994) The joint capsule: structure, composition, ageing and disease. J Anat 184:503–509 7928639 PMC1259958

[CR21] Zdanowicz U, Śmigielski R, Espejo-Reina A, Espejo-Baena A, Madry H (2016) Anatomy and vascularisation. Springer, Berlin Heidelberg

